# A review of methods used in assessing non-serious adverse drug events in observational studies among type 2 diabetes mellitus patients

**DOI:** 10.1186/1477-7525-9-83

**Published:** 2011-09-29

**Authors:** Liana Hakobyan, Flora M Haaijer-Ruskamp, Dick de Zeeuw, Daniela Dobre, Petra Denig

**Affiliations:** 1 Department of Clinical Pharmacology, University Medical Center Groningen, University of Groningen, The Netherlands; 2 Graduate School of Medical Sciences, University of Groningen, Groningen, The Netherlands

**Keywords:** non-serious adverse drug events, assessment methods, observational studies, type 2 diabetes mellitus

## Abstract

Clinical drug trials are often conducted in selective patient populations, with relatively small numbers of patients, and a short duration of follow-up. Observational studies are therefore important for collecting additional information on adverse drug events (ADEs). Currently, there is no guidance regarding the methodology for measuring ADEs in such studies. Our aim was to evaluate whether the methodology used to assess non-serious ADEs in observational studies is adequate for detecting these ADEs, and for addressing limitations from clinical trials in patients with type 2 diabetes mellitus. We systematically searched MEDLINE and EMBASE for observational studies reporting non-serious ADEs (1999-2008). Methods to assess ADEs were classified as: 1) medical record review; 2) surveillance by health care professionals (HCP); 3) patient survey; 4) administrative data; 5) laboratory/clinical values; 6) not specified. We compared the range of ADEs identified, number and selection of patients included, and duration of follow-up. Out of 10,125 publications, 68 studies met our inclusion criteria. The most common methods were based on laboratory/clinical values (n = 25) and medical record review (n = 18). Solicited surveillance by HCP (n = 17) revealed the largest diversity of ADEs. Patient surveys (n = 15) focused mostly on hypoglycaemia and gastrointestinal ADEs, laboratory values based studies on hepatic and metabolic ADEs, and administrative database studies (n = 5) on cardiovascular ADEs. Four studies presented ADEs that were identified with the use of more than one method. The patient population was restricted to a lower risk population in 19% of the studies. Less than one third of the studies exceeded pre-approval regulatory requirements for sample size and duration of follow-up. We conclude that the current assessment of ADEs is hampered by the choice of methods. Many observational studies rely on methods that are inadequate for identifying all possible ADEs. Patient-reported outcomes and combinations of methods are underutilized. Furthermore, while observational studies often include unselective patient populations, many do not adequately address other limitations of pre-approval trials. This implies that these studies will not provide sufficient information about ADEs to clinicians and patients. Better protocols are needed on how to assess adverse drug events not only in clinical trials but also in observational studies.

## Introduction

Medication safety assessment during the pre-approval regulatory phase is known to have limitations. Pre-approval clinical trials are often conducted in selective patient populations, with relatively small numbers of patients, and a short duration of follow-up [1,2]. Because of these limitations, several systems have been developed to monitor drug safety after marketing, including spontaneous reporting systems and risk management plans. Such safety assessment focuses primarily on detection of serious adverse drug events (ADEs) [3]. Little attention is given to the assessment of symptomatic or non-life-threatening ADEs, while the proportion of such ADEs is relatively common [4,5]. Symptomatic ADEs may affect patients' quality of life and adherence to treatment, and thereby the risk-benefit ratio of a drug.

Post-marketing observational studies are considered important to get more information on ADEs occurring in patient populations actually using the drugs [2,6,7]. This additional value, however, will only be achieved when the methodology used in such studies allows for adequate capturing of non-serious ADEs in an unrestricted population. The use of different methods for assessing ADEs, such as spontaneous and solicited reporting, medical record review, and patient surveys, may lead to differences in observed ADEs [8,9]. No guidance exists regarding the methods to be used for measuring ADEs in post-marketing studies [10-13].

Our aim was to evaluate the current methodology for assessing non-serious ADEs in observational studies, using oral antihyperglycemic drugs (OAD) as case. Research questions addressed are: (1) which methods of ADE assessment are used, (2) what is the range of non-serious ADEs captured for each method, (3) do the observational studies address known limitations of pre-approval trials regarding patient population and follow-up.

## Methods

### Search Strategy

We conducted a systematic search of MEDLINE and EMBASE for observational studies reporting on ADEs in patients with diabetes, and published between January 1 1999 and January 1 2009. We searched for papers using MeSH headings, subheadings and free-text terms related to the following domains: (1) "adverse events", and (2) "observational study design", and (3) "drug treatment" combined with "diabetes" (see Additional file 1 for detailed description of the search strategy). Using the boolean operator 'AND', only papers satisfying all three domains were included.

### Study Selection

Observational studies, i.e. non-experimental studies where decisions regarding the prescription of drugs to each patient were made by their health care provider in every-day clinical practice, were included when they reported rates of non-serious ADEs in adult patients with type 2 diabetes mellitus treated with OAD. We excluded open-label extensions of clinical trials. Non-serious ADEs were defined as any unfavourable and unintended sign (including abnormal laboratory values) or symptom or disease that may present during treatment with a pharmaceutical product and which was not life-threatening, requiring hospitalization or resulted in significant disability or death.

**Figure 1:**
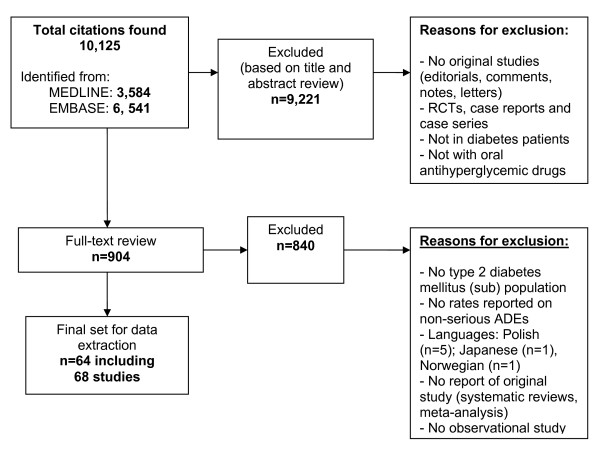
**Study flow diagram**.

The first title and abstract screening was done by LH, excluding editorials, comments, notes, letters, randomized clinical trials (RCTs), case reports, and studies not including patients with diabetes or not including OAD (see also Figure 1 for exclusions). PD screened a 10% sample which showed that LH had not excluded any potentially relevant studies. Screening of the remaining abstracts and full-texts was done by two reviewers independently. We restricted our selection to studies published in English, German, French, Spanish or Dutch language.

### Data Extraction

Information was collected from the selected publications each by two reviewers (PD/LH, DD/LH or FHR/LH) using a standardized data extraction form. Data were extracted regarding methods used for assessing ADEs, the ADEs identified, inclusion and exclusion criteria of patient population, sample size, and duration of follow-up. In addition, we extracted data on study design and medications covered. Discrepancies in data extraction occurred in 3 cases regarding 'methods used for assessing ADEs', in 8 cases regarding 'sample size', and 9 cases regarding 'duration of follow-up'. These discrepancies were often the result of unclear descriptions in the publications, and were solved by consensus based on a joint re-evaluation of what was described in the publication.

### Methods for ADE assessment

ADE assessment in observational studies can be based on review of existing practice-based data, such as medical records, laboratory reports, and administrative data, on surveillance by health care professionals (HCP) or on survey of patients [9,10,14]. Following this distinction, we defined the employed methods as: 1) medical record review, i.e. possible ADEs were collected from documentation or reports made by HCP in existing medical records; 2) solicited surveillance by HCP, i.e. requesting HCP to report possible ADEs either on Case Report Forms (prospective) or on socalled Prescription Event Monitoring forms (retrospective) [7]; 3) patient survey, including the use of open or closed patient questionnaires, checklists or diaries; 4) administrative data, making use of diagnostic codes related to possible ADEs in administrative or claims data; 5) laboratory or clinical values indicating ADEs, including results of laboratory measurements and physical examinations such as weight or blood pressure; 6) non-specified methods. Reported ADEs were categorized on anatomy or pathophysiology level according to Common Terminology Criteria for Adverse Events (CTCAE v3.0) classification [15].

### Patient population

Based on the reported patient inclusion and exclusion criteria, we classified studies as: (A) restricting the patient population to lower risk patients, (B) restricting to higher risk patients, (C) applying restrictions needed to achieve reliable outcome assessment, e.g. by excluding patients with a condition or medication use at baseline which would confound the outcome, (D) no restrictions reported.

### Sample size and duration of follow-up

We assessed the number of patients exposed to OAD, as well as the duration of their follow-up. For studies including more than one treatment group, we considered the sample size of the largest group exposed to OAD treatment. For studies including a diabetic subcohort, the overall number of exposed patients was considered as the sample size. Based on recommendations from regulatory agencies for safety assessment [11,12,16,17], we categorized sample sizes into six levels: 1) < 100 patients; 2) 100 to 299 patients; 3) 300 to 599 patients; 4) 600 to 1499 patients; 5) 1500 to 5000 and 6) > 5000 patients. Duration of follow-up for cohort studies was classified into: 1) ≤6 months; 2) 7-12 months; 3) 13 to 24 months; 4) more than 2 years.

### Data Analysis

Some publications reported on multiple studies with different patient populations and methods. We conducted analysis at this study level. We present the type, median number and interquartile range (IQR) of ADEs at category level reported for the six different methods of ADE assessment. Sample size and duration of follow-up are also compared for the different ADE assessment methods. We calculated the number of studies reaching regulatory recommendations for pre-approval safety assessment of drugs intended for long-term treatment of non-life-threatening conditions, i.e. 100 patients exposed for a minimum of 1 year or 300-600 patients for 6 months can be adequate to assess the pattern of ADEs over time [11,12].

## Results

The search resulted in 10,125 articles, out of which we selected 904 articles for full-text screening (Figure 1), resulting in 64 relevant articles reporting on 68 studies (see Additional file 2 for a description of the included studies).

### Methods of ADE assessment

The most commonly employed methods for assessing ADEs were based on laboratory/clinical values (n = 25), medical record review (n = 18), and solicited surveillance by HCP (n = 17) (Table 1). Surveillance by HCP was conducted prospectively using Case Report Forms in 12 studies, and retrospectively in 5 Prescription Event Monitoring studies. Among the 15 studies which used patient survey methods, 10 studies used a closed questionnaire, including two validated questionnaires [18,19], one used a checklist [20], one used a semi-structured interview guide where patients could report any perceived ADEs [21], and one used a 16-item content-validated questionnaire, containing closed and open-ended questions focusing among other issues on specific adverse events [22]. A patient diary was used in two studies [23,24]. Administrative databases were used in 5 studies, and in 7 studies, the data collection method was not fully specified.

**Table 1:** Median number and interquartile range (IQR) of different ADE categories identified for studies using different assessment methods

	Number of studies*	median number of ADE categories (IQR)	References
**Method of ADE assessment**			

Medical record review	18	2 (1-3)	[22,25,34,35,38,40,41,49-52,78-83]
Surveillance by HCP	17	4 (2-7)	[23,29,30,42,43,84-95]
Patient survey	15	1 (1-2)	[18-24,26,31,40,44,46,53,56]
Administrative data	5	1 (1-1)	[33,47,54,96,97]
Laboratory/clinical values	25	1 (1-2)	[25-28,32,35-37,39-41,44,45,50,55,57,80-83,98-101]
Non-specified	7	2 (1-10)	[27,28,36,48,99-101]

* Total exceeds 68 since one study may use several methods

### ADEs identified with different methods

The largest range of ADEs was identified with solicited surveillance by HCP, yielding a median of 4 ADE categories (Table 1). The range was even higher for retrospective surveillance (median 7, IQR 4-9) in comparison to prospective surveillance (median 3.5, IQR 2-6). Medical record review identified a median of 2 ADE categories (Table 1), covering many different areas (Table 2). Other specified methods assessed mostly 1 ADE category per study. Patient survey methods often focused on perceived hypoglycaemia or gastrointestinal ADEs (Table 2). Administrative databases were mainly used for cardiac ADEs, and laboratory/clinical values often included hepatic or metabolic problems or weight increase (Table 2). Four studies identified the same ADE, either hypoglycaemia or hepatic dysfunction, using more than one method, in particular a combination of laboratory values and other methods [25-28].

**Table 2:** Types of ADEs reported at category level for studies using different assessment methods (number of studies presented in table)

Adverse events at CTCAE category level	Medicalrecord review	HCP surveill-ance	Patient survey	Admini-strative data	Lab/clinical values	Non specified
Gastrointestinal	9	14	3			5
Neurology	3	6	1			3
Cardiac General	9	9		4	1	4
Blood/Bone Marrow	2	4			5	1
Pulmonary/Upper Respiratory	1	2				2
Hepatobiliary/Pancreas	3	7		1	11	2
Auditory/Ear		1				1
Ocular/Visual		1				
Dermatology/Skin	1	4				3
Musculoskelal/Soft Tissue		1				1
Renal/Genitourinary	1	1			2	2
Constitutional symptoms:						
- weight		6			12	
- other	1	3	1			
Pain	3	7	2			
Endocrine		1				
Infection		1				3
Allergy/Immunology		1				
Sexual/Reproductive Function	1					
Metabolic:						
- hypoglycaemia	7	7	8		3	5
- other		4	1		7	1
General ADEs/Tolerability*	3	12	3		3	5

CTCAE Common Terminology Criteria for Adverse Events v3.0; * not categorized

### Patient population

In 28 studies (41%), there were no specific limitations regarding the patient population included. In two studies (3%), no inclusion or exclusion criteria were specified [29,30]. Thirteen studies (19%) limited inclusion of patients to lower risk patients (category A) by including only patients with less severe diabetes [20,26,27,31-33] or patients on monotherapy [19,24,27,33-36], or OAD-naïve patients [27,35] or by excluding high risk patients who failed previous therapy [37] or with multiple comorbidity [20,38,39]. Fifteen studies (22%) limited the inclusion to more complicated cases (category B), such as inadequately controlled by or not tolerating previous medication [40-45], receiving combination treatment [46-48] or insulin [21,23,45,49] or treated with maximum dose of medication [50]. Furthermore, 18 studies (27%) excluded patients based on the presence at baseline of the outcome or a condition that could influence the outcome [18,24,25,33,37-39,47,51-55], non-availability of measurements and/or clinical visits [35,37,46,47,50,54,56,57], inability to fill in questionnaires (category C) [18,21,46,56].

### Sample size and duration of follow-up

**Figure 2:**
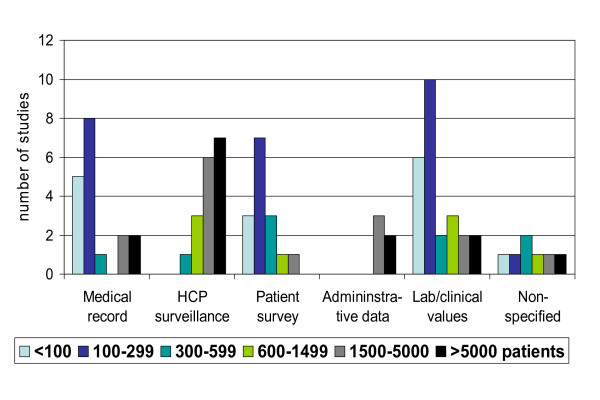
**Sample size included in studies using different assessment methods**.

Studies using patient survey methods, medical record review, or laboratory data often included less than 300 patients (Figure 2). A sample size of equal or more than 1500 was achieved by all studies using administrative databases, and in many studies using solicited surveillance by HCP. Overall, the follow-up period did not exceed one year in 77% of the cohort studies. Longer follow-up periods were mostly seen in studies using administrative data or laboratory/clinical values. Evaluation of sample size and follow-up jointly showed that all 3 cohort studies using administrative data exceeded the requirements of the guidelines for pre-approval safety assessment, whereas this was the case in less than a quarter of the studies using any of the other specified methods (Table 3).

**Table 3:** Number of cohort studies for each assessment method where sample size and follow-up period exceed regulatory recommendations for pre-approval safety assessment

		**Regulatory recommendations **[11,12]	
**Method of ADE assessment**	**Total number of cohort studies**	**> 100 patients > 12 months**	**> 300 patients > 6 months**

Medical record review	17	0	4
Surveillance by HCP	17	0	4
Patient survey	6	0	1
Administrative data	3	0	3
Laboratory/clinical values	22	3	3
Non-specified	7	2	1

Total	71	5	17

* Total exceeds 68 since one study may use several methods

## Discussion

Commonly used methods for assessing non-serious ADEs in patients with diabetes were based laboratory or clinical values, medical record review or solicited surveillance by HCP. The latter method identified the broadest range of ADE categories. Patient survey methods were used in 22% of the studies, and often focused on a limited range of ADEs, such as hypoglycaemia or gastrointestinal ADEs. The patient population was restricted to a lower risk population in a fifth of the studies. Less than one-third of studies exceeded pre-approval requirements regarding sample size and duration of follow-up.

Solicited surveillance by health care providers, using either prospective or retrospective data collection, revealed the largest diversity of ADEs, indicating that doctors register more events on such forms than in routine medical records. This is in line with previous findings that medical record review, although broadly used for assessing ADEs, results in incomplete findings [11,58]. Since there is no systematic documentation of ADEs in medical records, partly due to limitations of the documentation systems [59,60], review of such records lacks a standardized and reliable method to search for ADEs [61]. For non-serious, symptomatic ADEs the incomplete documentation of adverse events in medical records is even more the case when such ADEs do not warrant immediate action [1,62]. Prescription Event Monitoring studies, which make use of an open question to report all events that occurred during drug use on special forms, or prospective studies using prespecified Case Report Forms may solve this problem.

Patient reports can be of great value for ADE assessment because of the differences between reports from patients and health care providers [4,63-66]. Patients are a helpful source for the identification of many symptomatic ADEs, such as dizziness, malaise, fatigue, sexual function disorders, and pain [67-69]. Surprisingly, we found that patient survey methods were used in a relatively small number of studies, and moreover, often limited in their focus. Although comprehensive questionnaires have been developed to assess patient-perceived ADEs [70,71], such questionnaires were not used in observational studies for diabetes treatment.

Laboratory values may have a limited value for assessing non-serious ADEs, since mainly hepatic and metabolic problems were identified by these methods. This is in contrast with previous estimates that more than half of the ADEs can be detected by biochemical tests [72]. Administrative databases are also limited regarding the types of ADEs that can be identified. Such databases can be useful for assessing ADEs leading to hospitalization but have less value for assessing non-serious ADEs. Diagnostic administrative coding is likely to be both incomplete and unspecific for detecting non-serious ADEs [73], because these ADEs do not always call for a documented action from the health care provider [1,62]. Currently, European Medicines Agency regulators work on strengthening this source of information by establishing a European Network of Centres for Pharmacovigilance and Pharmacoepidemiology [74].

Combining methods for ADE assessment could address some limitations seen with all methods leading to under- or overreporting. ADEs which are likely to be underreported because of improper registration or coding in medical records might be complemented by laboratory values [73]. The same applies to doctor and patient reports that may complement each other [75]. In our review, however, only a four studies identified the same ADE using a combination of methods.

Observational post-marketing studies can provide additional information on ADEs when sufficient numbers of patients are being followed in daily practice, including those with higher risks, more comorbidity, concomitant drugs, and longer disease duration. The majority of studies in our review included such patient populations, thus adding valuable information on ADEs in patient groups underrepresented in pre-approval trials. The number of patients included and the duration of follow-up, however, showed similar limitations as pre-registration trials, and the majority of studies did not go beyond the pre-approval recommendations for safety assessment of diabetes medication. Because of workload, long follow-up for large numbers of patients can be problematic in studies where the patients or the health care providers need to provide the information. It is less problematic when data can be collected from existing databases.

Our study has some limitations. It has previously been recognized that searching the literature for studies reporting on drug safety is difficult [76,77], and also indexing of observational studies may not be as robust as of RCTs. We therefore used a broad search strategy to identify possibly relevant studies. Second, the results are based on studies conducted in diabetes patients using OADs. For other therapeutic areas and other drugs, results may be different. Third, we used the CTCAE v3.0 classification to define ranges of ADEs identified by different methods. Although the CTCAE categories are quite similar to the primary system organ classes in the MedDRA hierarchy, minor differences in ranges may occur when using this alternative classification system. Finally, we encountered several problems regarding unclear or incomplete reporting. Although it was not our aim to evaluate studies on the quality of reporting, and we did not exclude studies on these grounds, we observed that information on, for example, exclusion criteria and response rates was often lacking.

## Conclusion

The current set up of ADE assessment in post-marketing studies is not adequate for countering limitations acknowledged in pre-approval trials. The assessment of non-serious ADEs is limited by the choice of methods. Many observational studies rely on methods that are inadequate for identifying all possible ADEs. Patient survey methods are underutilized, and there is a lack of studies that try to combine different methods to assess ADEs. This implies that these studies will not provide sufficient information about ADEs to clinicians and patients. Better protocols are needed on how to assess adverse drug events not only in clinical trials but also in observational studies.

## List of abbreviations

ADEs: adverse drug events; CTCAE v3.0: Common Terminology Criteria for Adverse Events version 3.0; HCP: health care provider; IQR: interquartile range; OAD: oral antihyperglycemic drugs; RCTs: randomized clinical trials.

## Competing interests

The authors declare that they have no competing interests.

## Authors' contributions

LH conducted the literature search, participated in the data extraction and analysis, and drafted the manuscript. FHR conceived of the study, and participated in its design and in the data extraction and analysis. DdZ participated in the conception and design of the study. DD participated in the data extraction and analysis. PD participated in the conception and design of the study, in the data extraction and analysis, and edited the final manuscript. All authors read and approved the final manuscript.

## Supplementary Material

Additional file 1 **Search strategy used for eligible studies**. Provides the domains, terms and boolean operators used in the systematic search of Medline and Embase for observational studies reporting on ADEs in patients with diabetes. Click here for file

Additional file 2 **Description of the studies included in the review**. Provides the following data for each included study: data collection method employed for ADE assessment, publication year, country, study design, type of ADEs included, sample size, follow up period, patients selection. Click here for file
