# Supplementary material for: A review of methods used in assessing non-serious adverse drug events in observational studies among type 2 diabetes mellitus patients
Source: Health Qual Life Outcomes. 2011 Sep 29;9:83. doi: 10.1186/1477-7525-9-83 (PMC3198877; doi:10.1186/1477-7525-9-83)
Supplement: Additional file 1 — Search strategy used for eligible studies. Provides the domains, terms and boolean operators used in the systematic search of Medline and Embase for observational studies reporting on ADEs in patients with diabetes. [file 1477-7525-9-83-S1.PDF]

Search strategy used for eligible studies
Boolean operator 'AND' was used to search for combination of Domains (1: adverse events) AND (2: observational study design) AND (3: drug treatment AND diabetes); operator 'OR' was used to include all headings and terms within each domain.

	Domains	MeSH	Subheading	Free text terms
1	Adverse events	1. exp Drug Toxicity/	1. adverse effects.fs.<br><br>2. chemically induced.fs	(safety or side effect$ or adverse drug event$ or adverse drug reaction$ or drug complication$ or adverse effect$ or ADR$ or medic$ error$ or complication$ or adverse medical event$ or medication symptom$ or drug-drug interaction or medication-attributed barrier$ or patient-important outcome$ or toxic effect$ or toxicity or drug related morbidity or medication-related problem$ or tolera$ or risk).tw.
2	Observational study design	1. exp Product Surveillance, Postmarketing<br><br>2. Drug Monitoring/<br><br>3. Risk Assessment/<br><br>4. exp Data Collection/		(prescription-event monitoring or prescription event monitoring or PEM or post-marketing surveillance$ or post marketing product surveillance$ or postmarketing surveillance$ or postmarketing evaluation stud$ or post-marketing evaluation stud$ or post marketing evaluation stud$ or postmarketing drug surveillance$ or post-marketing drug surveillance$ or post marketing drug surveillance$ or cross sectional stud$ or cross-sectional stud$ or survey$ or case-control or cohort stud$ or prospective cohort or retrospective cohort or precrib$ or prescript$ or risk perception or follow-up stud$ or follow up stud$ or record$ review or chart review or voluntary report$ or spontaneous report$ or intensive monitoring or electronic record$ or questionnaire$ or report$ or self-report$ or patient-report$ or subjectively-report$ or withdraw$).tw.
3	Drug treatment	exp Drug Therapy/	Drug therapy. Fs.	(drug or pharmacotherap$ or polypharmacy).tw
	Diabetes	exp Diabetes Mellitus/		(diabet$).tw.
