# Supplementary material for: A review of methods used in assessing non-serious adverse drug events in observational studies among type 2 diabetes mellitus patients
Source: Health Qual Life Outcomes. 2011 Sep 29;9:83. doi: 10.1186/1477-7525-9-83 (PMC3198877; doi:10.1186/1477-7525-9-83)
Supplement: Additional file 2 — Description of the studies included in the review. Provides the following data for each included study: data collection method employed for ADE assessment, publication year, country, study design, type of ADEs included, sample size, follow up period, patients selection. [file 1477-7525-9-83-S2.DOC]

**Description of the studies included in the review** (see for abbreviations, at bottom of table)

| Reference | **Data collection method** | **Study (publication year)** | **Country** | **Study design** | **Type of ADEs included** | **Sample size (range for subpopulations)** | **Follow-up period*** | **Patients selection** |
| --- | --- | --- | --- | --- | --- | --- | --- | --- |
| **Health care provider method**s | | |  |  |  |  |  |  |
| [38] | MedRec | Blonde et al. (2004) | US | RC | GI, P | 468  (158-310) | 12 | A, C |
| [34] | MedRec | Chokrungvaranon et al. (2007) | US | RC | GI, D/S | 92 | 6 | A |
| [78] | MedRec | Donekal and Shomali (2008) | US | RC | GI, M(h/g) | 81 | 6 | D |
| [40] | MedRec | Feher et al. (2007) | UK, Wirral | RC | GI | 28 | 5.9 | B |
| [40] | MedRec | Feher et al. (2007) | UK, London | RC | GI | 21 | 6 | B |
| [49] | MedRec | Marceille et al. (2004) | US | RC | CV | 139 | 6 | B |
| [51] | MedRec | Maru et al. (2005) | UK | RC | CV | 25690  (4107-11350) | 30 | C |
| [52] | MedRec | Nichols et al. (2005) | US | RC | CV | 8063  (272-1834) | 72 | C |
| [79] | MedRec | Redondo-Capafons et al. (2005) | Spain | PC | GI, R/G, M(h/g) | 135 | 10 days | D |
| [22] | MedRec | Yusuff et al. (2008) | Nigeria | CS | N, Pul, P, S/R, M(h/g), AE/T | 200 |  | D |
| [35] | MedRec+L(r) | Asche et al. (2008) | US | RC | GI, N, CV, P ,M, M(h/g), AE/T + C(w), H | 5438  (889-2326) | 13 | A, C |
| [80] | MedRec+L(p) | Burk et al. (2004) | US | PC | GI, N, C, CV + C(w), H | 362  (101-261) | 11 | D |
| [41] | MedRec+L(r) | Hussein et al. (2004) | Australia | RC | CV, M(h/g), AE/T + C(w), H, M | 203  (96-107) | 12 | B |
| [25] | MedRec+L(r) | Jick et al. (1999) | UK | RC | H + H | 40190  (175-22691) | 3 | C |
| Reference | **Data collection method** | **Study (publication year)** | **Country** | **Study design** | **Type of ADEs included** | **Sample size (range for subpopulations)** | **Follow-up period*** | **Patients selection** |
| [50] | MedRec+L(r) | King and Armstrong (2002) | US | RC | CV, B + M(h/g), C(w), H | 100 | 3 | B, C |
| [81] | MedRec+L(r) | Manley and Allcock (2003) | US | RC | CV, B + C(w) | 40 | 3 | D |
| [82] | MedRec+L(r) | Swislocki et al. (1999) | US | RC | GI, M(h/g) + M | 251 | 9 | D |
| [83] | MedRec+L(r) | Tang et al. (2003) | US | RC | CV + C(w) | 115 | 12 | D |
| [84] | Surveillance by HCP | Fehmann von (2001) | Germany | PC | GI, N, A/I, D/S, AE/T | 2654 | M | D |
| [42] | Surveillance by HCP | Hung et al. (2006) | Taiwan | PC | GI, P, AE/T | 1558 | 3.5 | B |
| [85] | Surveillance by HCP | Kane et al. (2004) | US | PC | GI, N, C(w), CV, H, P | 316 | 6 | D |
| [86] | Surveillance by HCP | Kawamori et al. (2007) | Japan | PC | H, CV | 24993 | 18 | D |
| [23] | Surveillance by HCP +P/d | Klocke et al. (2003) | Germany | PC | GI, P, AE/T + M(h/g) | 1142 | 3 | B |
| [30] | Surveillance by HCP | Landgraf et al. (2000) | Germany | PC | M(h/g), AE/T | 5985 | 1.5 | n.s. |
| [87] | Surveillance by HCP | Mertes (2001) | Germany | PC | GI, C(w), H, P, M(h/g), AE/T | 1954 | 60 | D |
| [88] | Surveillance by HCP | Pan and Landen (2007) | China | PC | GI, AE/T | 2248 | 3 | D |
| [89] | Surveillance by HCP | Rosak et al. (2005) | Germany | PC | C(w), CV, H, B, M, M(h/g), AE/T | 11014 | 6 | D |
| [90] | Surveillance by HCP | Scholz et al. (2001) | Germany | PC | E, GI, N, Pul, CV, A/E, D/S, Ms, R/G, B, M, M(h/g), AE/T | 22045 | 2 | D |
| [91] | Surveillance by HCP | Slama et al. (2008) | France | PC | GI, C(w), CV, H, B | 3580 | 12 | D |
| [92] | Surveillance by HCP | Spengler et al. (2005) | Germany | PC | GI, H, M(h/g), AE/T | 26044 | 3 | D |
| Reference | **Data collection method** | **Study (publication year)** | **Country** | **Study design** | **Type of ADEs included** | **Sample size (range for subpopulations)** | **Follow-up period*** | **Patients selection** |
| [29] | Surveillance by HCP | Biswas et al. (2001) | UK | RC | GI, C, CV, H | 1344 | 6 | n.s. |
| [93] | Surveillance by HCP | Kasliwal et al. (2008) | UK | RC | GI, N, C(w), CV, O/V, H, P, B, M, AE/T | 12772 | 8 | D |
| [94] | Surveillance by HCP | Kubota et al. (2001) | Japan | RC | GI, C(w), CV, H | 3115  (272-880) | 6 | D |
| [95] | Surveillance by HCP | Marshall et al. (2006) | UK | RC | GI, N, C, I, D/S, P, M(h/g), AE/T | 5731 | 6 | D |
| [43] | Surveillance by HCP | Twaites et al. (2007) | UK | RC | GI, N, C, Pul, CV, D/S, P, M, M(h/g), AE/T | 4557 | 6 | D |
| **Patient oriented methods** (some with additional Lab) | | |  |  |  |  |  |  |
| [46] | PQc(n/s) | Alvarez Guisasola et al.(2008) | Various^a^ | CS | M(h/g) | 1709 |  | B, C |
| [31] | PQc(n/s) | Chao et al. (2007) | US | CS | AE/T | 445 |  | A |
| [56] | PQc(n/s) | Grant et al. (2003) | US | CS | AE/T | 128 |  | C |
| [40] | PQc(n/s)+L(p) | Feher et al. (2007) | UK, Liverpool | PC | GI+C(w) | 22 | 3 | B |
| [40] | PQc(n/s)+L(p) | Feher et al. (2007) | UK, Isle of Wight | PC | GI+C(w) | 24 | 4.7 | B |
| [44] | PQc(n/s)+L(p) | Hershon and Herson (2000) | US | PC | M(h/g)+C(w), H | 207  (6-42) | 12 | B |
| [18] | PQc(s) | Bytzer et al. (2001) | Australia | CS | GI, P | 405  (2-195) |  | C |
| [53] | PQc(s) | Miller et al. (2001) | US | CS | M(h/g) | 1055  (13-177) |  | C |
| [19] | PQc(s) | Woodcock et al. (2007) | UK | CS | M, M(h/g) | 131 |  | A |
| [26] | PQc(s)+L(p) | UK Hypoglycaemia Study Group (2007) | UK | PC | M(h/g) + M(h/g) | 274  (108) | 9.5 | A |
| [20] | P/ch | Vexiau et al. (2008) | France | CS | M(h/g) | 400 |  | A |
| [22] | PQ/o/c | Yusuff et al. (2008) | Nigeria | CS | N, C, P | 200 |  | D |
| Reference | **Data collection method** | **Study (publication year)** | **Country** | **Study design** | **Type of ADEs included** | **Sample size (range for subpopulations)** | **Follow-up period*** | **Patients selection** |
| [21] | PQo | Haugbolle and Sorensen (2006) | Denmark | CS | AE/T | 192 |  | B, C |
| [24] | P/d | Guagnano et al. (2000) | Italy | PC | M(h/g) | 340 | 11 | A, C |
| **Administrative data** | | |  |  |  |  |  |  |
| [96] | Administrative | Casscells et al. (2008) | US | CS | CV | 231962 |  | D |
| [47] | Administrative | Delea et al. (2003) | US | RC | CV | 33544  (5441-28103) | 40 | C,B |
| [33] | Administrative | McAlister et al. (2008) | Canada | RC | CV | 5631  (1469-4162 | 56 | A,C |
| [97] | Administrative | Rajagopalan et al. (2005) | US | RC | H | 8916  (1137-1847) | 10 | D |
| [54] | Administrative | Shaya et al. (2005) | US | RC | CV | 2756  (677-2079) | M | C |
| **Laboratory/ clinical values or non-specified methods** | | | |  |  |  |  |  |
| [55] | L(p) | Filioussi et al. (2003) | Greece | CS | B | 600 |  | C |
| [39] | L(p) | Hermann et al. (2004) | Sweden | CS | B | 84 (31-53) |  | A, C |
| [32] | L(p) | Karagiannis et al. (2008) | Germany | PC | M, AE/T | 1170 | 5 | A |
| [98] | L(p) | Monster et al. (2003) | The Netherlands | CS | R/G | 7365 |  | D |
| [57] | L(r) | Chalasani et al. (2005) | US | RC | H | 838 | 12 | C |
| [45] | L(r) | Furlong et al. (2002) | UK | RC | C(w) | 200  (67-133) | 29 | B |
| [37] | L(r) | Olansky et al. (2003) | US | RC | C(w) | 1115  (78-133) | 7 | A, C |
| [99] | N/sp+L(p) | Abbasi et al. (2000) | US | PC | AE/T + M | 110 | 24 | D |
| [100] | N/sp+L(p) | Gavin et al. (2000) | US | PC | GI + H | 40 | 12 | D |
| [36] | N/sp+L(p) | Hanefeld et al. (2006) | Germany | PC | CV, M(h/g) + C(w) | 500  (250-250) | 42 | A |
| [27] | N/sp+L(p) | Taki et al. (2005a) | Japan | PC | GI, N, I, CV, A/E, D/S, M(h/g), AE/T + H, B, M, M(h/g), AE/T | 525 | 3 | A |
| Reference | **Data collection method** | **Study (publication year)** | **Country** | **Study design** | **Type of ADEs included** | **Sample size (range for subpopulations)** | **Follow-up period*** | **Patients selection** |
| [28] | N/sp+L(p) | Taki et al. (2005b) | Japan | PC | GI, N, I, CV, Pul, D/S, Ms, R/G, H, M(h/g), AE/T + CV, H, B, R/G, M, M(h/g), AE/T | 3150 | 3 | D |
| [101] | N/sp+L(p) | Taki et al. (2006) | Japan | PC | GI, N, I, CV, Pul, D/S, R/G, H, M, M(h/g), AE/T + H, B, M | 993 | 15 | D |
| [48] | N/sp | Schatz et al. 2003 | Germany | PC | GI, M(h/g), AE/T | 11476 | 3.2 | B |

**Abbreviations used for Description of the studies included in the review**

| **Study design:** CS=cross-sectional; PC=prospective cohort; RC=retrospective cohort |
| --- |
| **Data collection method:** MedRec: medical record review; L(r)=laboratory/clinical values (routine); L(p)=laboratory/clinical values (planned, pre-specified); PQc(n/s)=patient closed questionnaire, non-specific; PQc(s)=patient closed questionnaire, specific; PQo=patient open questionnaire; PQ/o/c=patient questionnaire with both open and closed questions; P/ch=patient /checklist; P/d=patient diary; N/sp=non-specified;; |
| **Adverse Drug Events (ADEs):** E=endocrine, GI=gastrointestinal, N=neurology, C=constitutional, C(w)=constitutional (weight); I=infections, Pul=pulmonary, CV=cardiac, A/I=allergy/immunology, O/V=ocular/visual, A/E=auditory/ear, D/S=dermatology/skin, Ms=musculoskeletal, H=hepatobiliary, R/G=renal/genitourinary, P=(abdominal)pain, S/R=sexual/reproductive function, B=blood/bone marrow, M=metabolic, M(h/g)=hypoglycemia; AE/T=general ADEs or tolerability |
| **Sample size**: total number of patients exposed to drug in study; range is lowest-highest number of patients per treatment arm |
| **Follow-up period**:* in months (mean), only for cohort studies; M=missing data |
| **Patient selection (inclusion/exclusion criteria):** A=restricting patient population to lower risk patients, B=restricting to higher risk patients, C=applying restrictions needed to achieve reliable outcome assessment, D=no restrictions (reported), n.s.=no specifications provided on patient population |
